# Supplementary material for: Interactions between Mediterranean Diet Supplemented with Dairy Foods and the Gut Microbiota Influence Cardiovascular Health in an Australian Population
Source: Nutrients. 2023 Aug 19;15(16):3645. doi: 10.3390/nu15163645 (PMC10459086; doi:10.3390/nu15163645)
Supplement: Supplementary file 1 [file nutrients-15-03645-s001.zip › nutrients-2560303-supplementary.pdf]

# Interactions between Mediterranean Diet Supplemented with Dairy Foods and the Gut Microbiota Influence Cardiovascular Health in an Australian Population

Jocelyn M. Choo[1,2], Karen J. Murphy[3,4], Alexandra T. Wade[3,5], Yanan Wang[6], Ella L. Bracci[3,4], Courtney R. Davis[3,4], Kathryn A. Dyer[4], Richard J. Woodman[7], Jonathan M. Hodgson[8,9], Geraint B. Rogers[1,2]

[**] Jocelyn M Choo and Karen J Murphy are joint first authors

## SUPPLEMENTARY TABLE AND FIGURES

**Supplementary Table S1. Taxa that are significantly altered by the 8-week MedDairy (MD) or low-fat control diet (LFD) intervention.**

| Bacteria | Within group comparison | | | | Between group comparison | |
|---|---|---|---|---|---|---|
| | MedDairy (MD) | | Low-fat diet (LFD) | | 8-weeks | |
| | p | FDR | p | FDR | p | FDR |
| Lachnospiraceae NK4A136 | 0.011* | 0.754 | 0.748 | 1 | 0.266 | 1 |
| Streptococcus | 0.017* | 0.754 | 0.216 | 0.858 | 0.078 | 1 |
| Collinsella | 0.021* | 0.754 | 0.003* | 0.461 | 0.014* | 0.977 |
| Butyricicoccus | 0.022* | 0.754 | 0.620 | 1 | 0.050* | 0.982 |
| Oscillospirales (uncultured) | 0.014* | 0.754 | 1 | 1 | 0.505 | 1 |
| Oscillospirales UCG-002 | 0.031* | 0.897 | 0.405 | 0.858 | 0.659 | 1 |
| Veillonella | 0.036* | 0.932 | 0.724 | 1 | 0.049* | 0.982 |
| Ruminococcaceae UBA1819 | 0.043* | 0.966 | 0.415 | 0.858 | 0.110 | 0.982 |
| Oscillospirales UCG-010 | 0.461 | 0.966 | 0.012* | 0.720 | 0.572 | 1 |
| Oscillospirales UCG-005 | 0.727 | 1 | 0.036* | 0.858 | 0.074 | 0.982 |
| Moryella | 0.624 | 1 | 0.014* | 0.719 | 0.059 | 0.982 |
| Izemoplasmatales | 0.820 | 1 | 0.026* | 0.858 | 0.148 | 0.982 |
| Holdemania | 0.913 | 1 | 0.031* | 0.858 | 0.746 | 1 |
| Rikenellaceae RC9 gut group | 0.944 | 1 | 0.033* | 0.858 | 0.155 | 0.982 |

Statistical analyses were performed using the Wilcoxon sign-rank test for within group comparison and the Mann-Whitney test for between group comparison. * denotes p≤ 0.05.

**Supplementary Table S2. Correlation analysis between *Butyricicoccus* and clinical, anthropometric and cognitive measurements for both MedDairy (MD) and low-fat diet (LFD) groups combined (whole dataset), and stratified by MD or LFD diet.**

| Variable 1 | Variable 2 | MD and LFD groups combined | | |
|---|---|---|---|---|
| | | r | p | FDR |
| 10-point MDS | *Butyricicoccus* | 0.283 | 0.003* | 0.048* |
| 18-point MDS | *Butyricicoccus* | 0.331 | <0.001* | 0.011* |
| SBP (clinic) | *Butyricicoccus* | -0.241 | 0.013* | 0.114 |
| Glucose | *Butyricicoccus* | 0.228 | 0.019* | 0.152 |
| 10-point MDS | *Colinsella* | -0.091 | 0.353 | 0.747 |
| 18-point MDS | *Colinsella* | -0.301 | 0.002* | 0.028* |
| Cholesterol:HDL | *Colinsella* | 0.215 | 0.027* | 0.195 |

| Variable 1 | Variable 2 | MD only | | | LFD only | | |
|---|---|---|---|---|---|---|---|
| | | r | p | FDR | r | p | FDR |
| 10-point MDS | *Butyricicoccus* | 0.464 | 0.006* | 0.094 | 0.199 | 0.246 | 0.694 |
| 18-point MDS | *Butyricicoccus* | 0.509 | 0.002* | 0.050* | 0.196 | 0.252 | 0.704 |
| SBP (clinic) | *Butyricicoccus* | -0.377 | 0.026* | 0.232 | -0.203 | 0.235 | 0.691 |
| Glucose | *Butyricicoccus* | 0.396 | 0.019* | 0.192 | 0.070 | 0.683 | 0.967 |
| 10-point MDS | *Colinsella* | -0.165 | 0.350 | 0.771 | -0.065 | 0.705 | 0.967 |
| 18-point MDS | *Colinsella* | -0.329 | 0.058 | 0.354 | -0.098 | 0.570 | 0.914 |
| Cholesterol:HDL | *Colinsella* | 0.183 | 0.293 | 0.539 | 0.341 | 0.042* | 0.328 |

r denotes correlation coefficient; FDR denotes false discovery rate adjustment multiple testing.

**A**

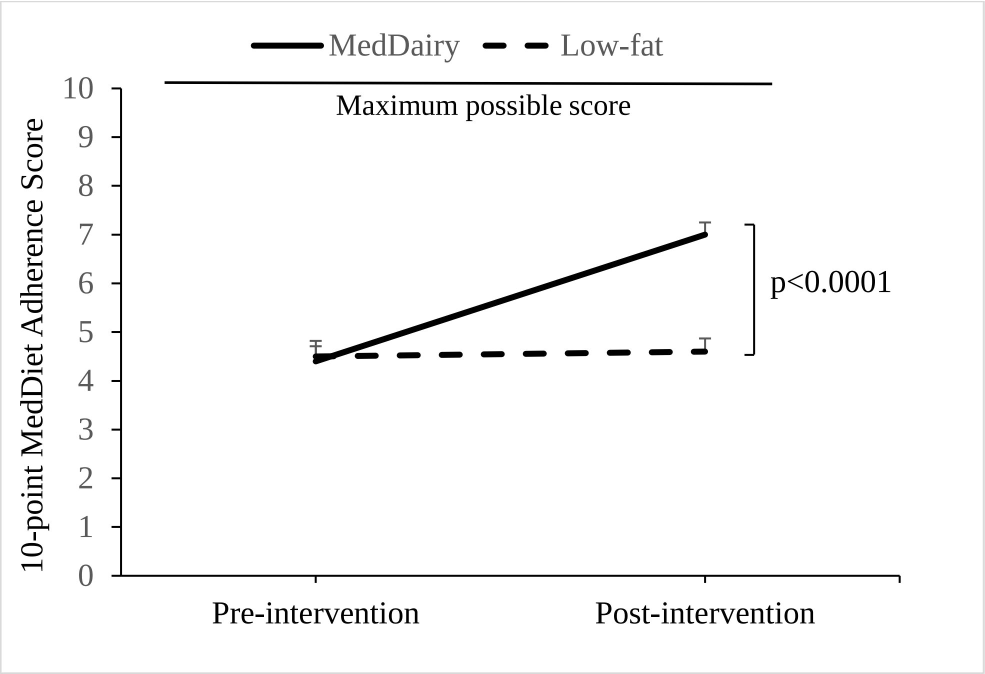


**B**

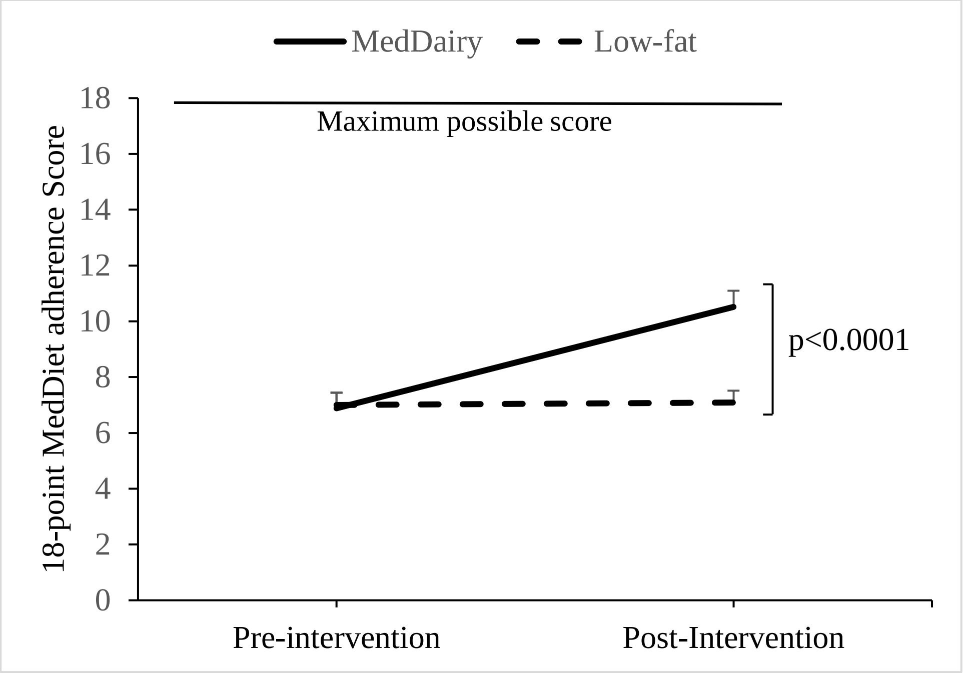


**Supplementary Figure S1**. (A) Mediterranean diet score before and during MedDairy and Low-Fat dietary phases. Maximum possible score = 10 based on Trichopoulou *et al.* (2003). (B) Mediterranean diet score before and during MedDairy and Low-Fat dietary phases. Maximum possible score = 18 based on Sofi *et al.* (2014). Data is shown as Mean ± standard error of mean. p<0.0001 indicate significant difference between groups.

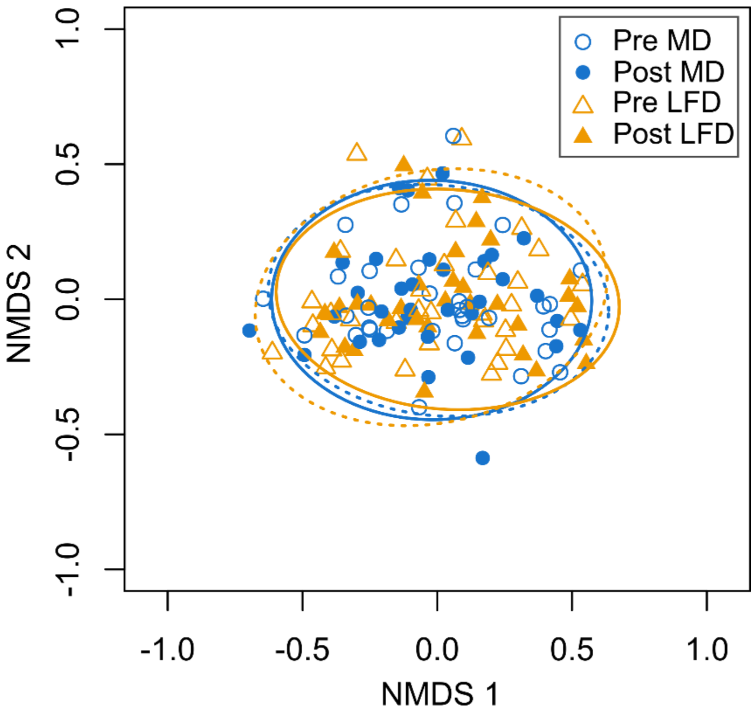


**Supplementary Figure S2.** Non-metric multidimensional scaling (NMDS) ordination plot based on the gut microbiota composition before and after 8-weeks of MedDairy (MD) or low-fat diet (LFD) intervention.

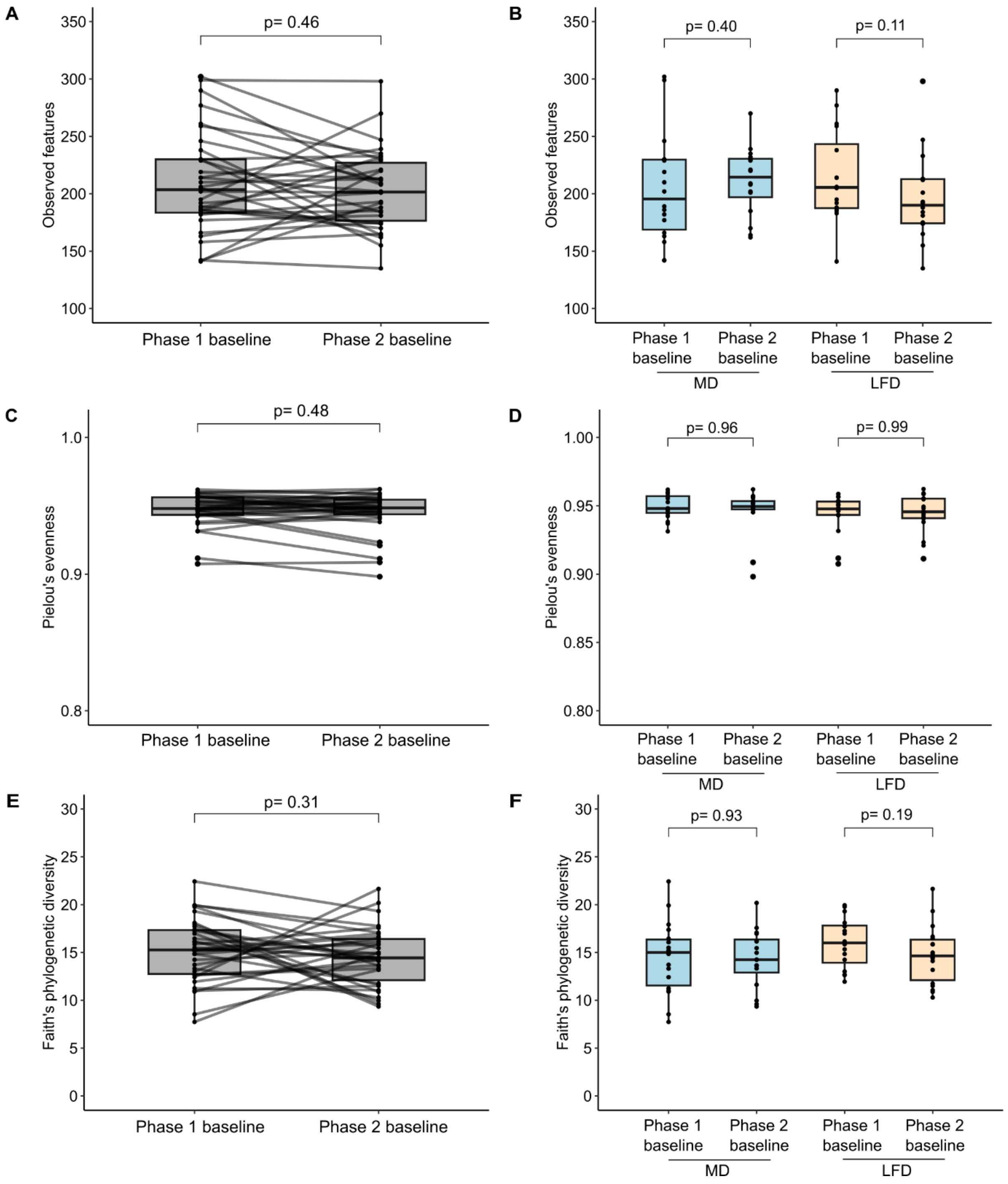


**Supplementary Figure S3.** Comparison of microbiota characteristics between the baseline of Phase 1 and Phase 2 for alpha diversity measures of (A, B) microbial richness, (C, D) evenness and (E, F) diversity. Statistical comparisons were performed using the Wilcoxon sign-rank test (within-group assessment) or Mann-Whitney test (between group assessment).

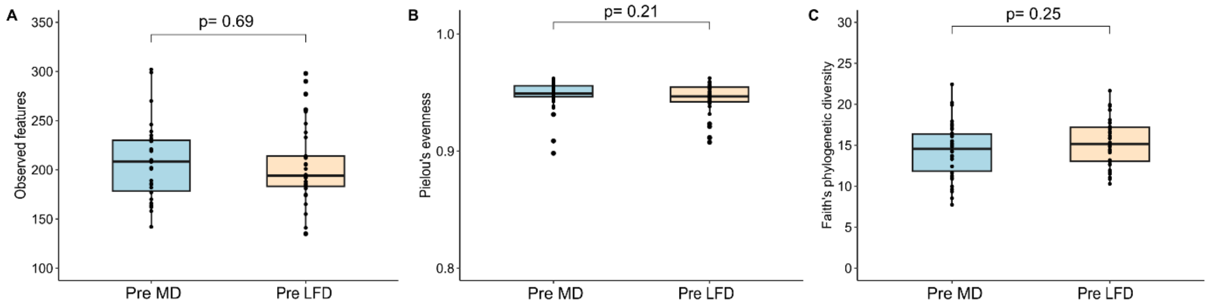


**Figure S4.** Comparison of microbiota characteristics between the MedDairy (MD) and Low-fat diet (LFD) groups at baseline for alpha diversity measures of (A) microbial richness, (B) evenness and (C) diversity. Statistical comparisons were performed using the Mann-Whitney test (between group assessment).

**REFERENCES**

1. Trichopoulou A, Costacou T, Bamia C, et al. Adherence to a Mediterranean diet and survival in a Greek population. *The New England journal of medicine* 2003;348(26), 2599-608.

2. Sofi F, Macchi C, Abbate R, et al. Mediterranean diet and health status: an updated meta-analysis and a proposal for a literature-based adherence score. *Public Health Nutr* 2014;17(12), 2769-82.
